# Relapsing Ipsilateral Vestibular Neuritis

**DOI:** 10.1155/2017/3628402

**Published:** 2017-12-04

**Authors:** Duilio Emiliano De Schutter, Nicolás Pérez Fernández

**Affiliations:** ^1^Department of Neurology, Universidad Nacional de Cuyo, Mendoza, Argentina; ^2^Department of Otorhinolaryngology, Clínica Universidad de Navarra, Pamplona, Spain

## Abstract

In 2013, a 70-year-old male was admitted with an acute episode of vertigo, nausea, and vomiting with duration of one day. The patient's background included prehypertension, vitiligo, left ventricular hypertrophy, and Sjögren's syndrome. He denied any previous episode of vertigo or migraine manifestations. Neither hearing loss nor tinnitus or otorrhea was detected at the time of evaluation. No neurological symptoms were found. There was a left-beating spontaneous nystagmus Grade 3. The patient could stand still and walk on his own with some help without falling. Day 1 vHIT showed a significant reduction in VOR gain and refixation saccades after head impulses were delivered in the planes of the right anterior and horizontal semicircular canals. MRI showed no significant findings. He was treated with steroids. A vHIT performed 14 days later showed recovery of gains and no refixation saccades. In 2015, the patient had a new episode of acute vertigo. The clinical examination was similar, and the vHIT revealed a new drop of right superior and lateral canal gains. Cervical and ocular VEMPs were performed, and no significant asymmetry was detected. Serum PCR for herpes viruses resulted negative. Contrast MRI was performed without relevant brain findings.

## 1. Introduction

Vestibular neuritis (VN) is the sixth cause of vertigo with an incidence of 8% according to large population studies [1, 2]. Residual vestibular symptoms are frequent after a VN episode. However, BPPV (about 15% of patients after neuritis) remains the main cause of these symptoms [3, 4]. Only 2% of VN patients are likely to suffer a new episode in the other ear, whereas ipsilateral relapse (or recurrence) is even rarer [5, 6]. Previous studies with less individuals reported larger incidences [7]. Nevertheless, considering Bell's palsy as an equivalent disease, it is known that this entity could have many recurrences. Thus, it could be possible that the same patient experiences multiple episodes. Etiologic factors have not been elucidated, but there are no arguments in favor of other etiologies other than viral [8–12]. There are no reports of relapses of vestibular neuritis assessed by vHIT.

## 2. Results

**Figure 1:**
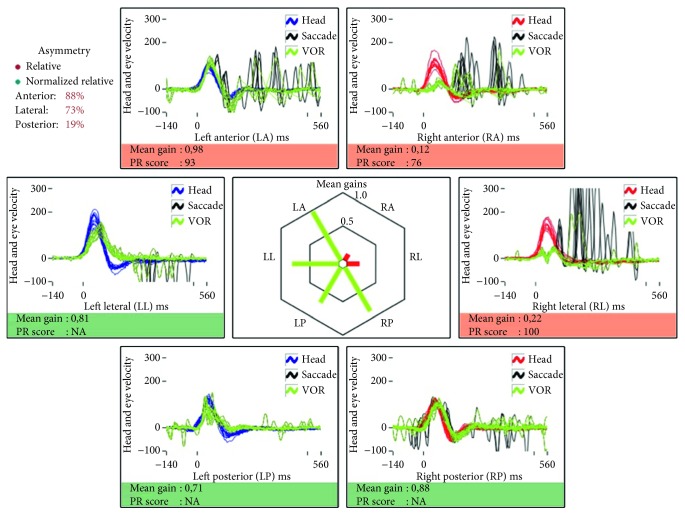
vHIT three days after symptoms begun on first episode.

**Figure 2:**
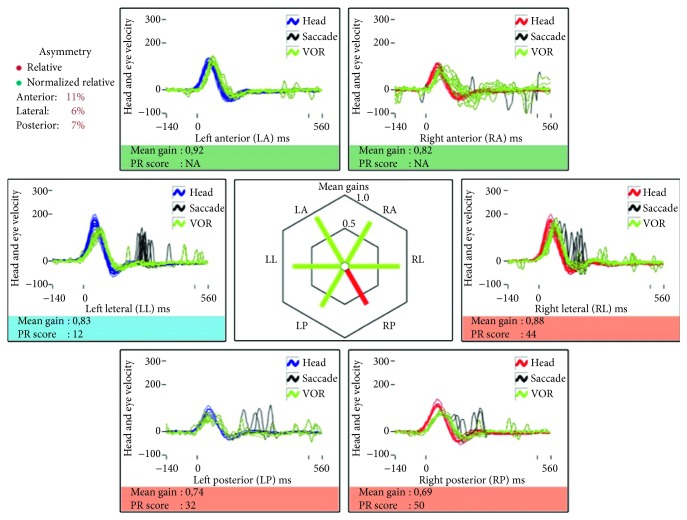
vHIT 14 days after first episode.

In April 2013, a 70-year-old male patient with a spontaneous acute vestibular syndrome was admitted to the clinic 24 hours after the onset of symptoms: these were continuous, not evoked by any positional change, and in that period of time, no other symptoms were revealed. He denied suffering from hearing loss, tinnitus, pressure in the ear, or headache. During the examination, there was a first-degree, spontaneous, left-beating nystagmus without visual fixation, which became a third-degree nystagmus using Frenzel goggles. There was no ocular misalignment, and the ocular tilt was negative. The patient was able to stand without help and needed aid to walk. Upon bedside testing, there were clear refixation saccades for rightward head impulses. He was then diagnosed with a right-side vestibular neuritis. A vHIT showed decreased gains when the superior and horizontal semicircular canal receptors were tested on the right side (0.34 and 0.58, resp.) with several refixation saccades (Figure 1). A diagnosis of vestibular neuritis affecting the superior subdivision of the nerve was then given. The patient was admitted and treated with oral steroids (prednisone 60 mg/d) for seven days; he clinically recovered, and a second vHIT (two days after the first) still showed decreased gains in the affected canals of 0.12 and 0.22, respectively. An MRI performed 24 hours later showed normal brainstem and posterior fossa with no signs of an internal canal tumor. The fourth day, he was sent home with a plan of progressive decrease of steroid dose. A third vHIT performed 14 days later (when clinical recovery was almost complete) showed gain recovery in the right side of 0.83 in the superior semicircular canal and 0.88 in the horizontal canal (with clear covert refixation saccades), and a nonsignificant gain decrease in the posterior canal was detected (Figure 2). At that time, no spontaneous nystagmus was observed (with and without vision suppression), not even after head-shaking.

**Figure 3:**
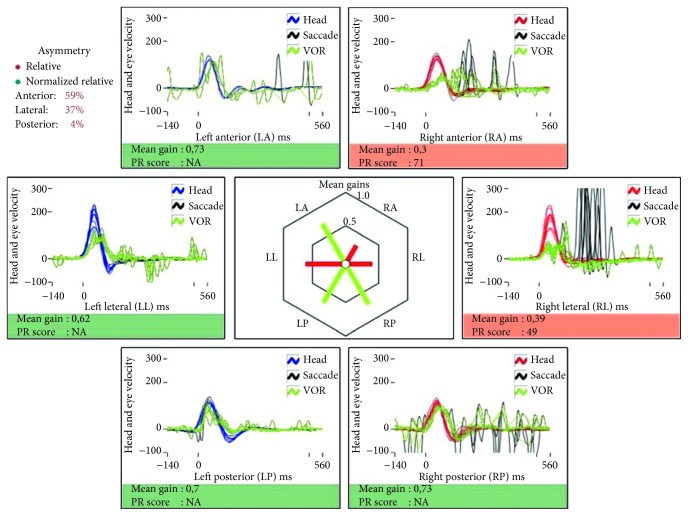
vHIT three days after the beginning of the second episode.

**Figure 4:**
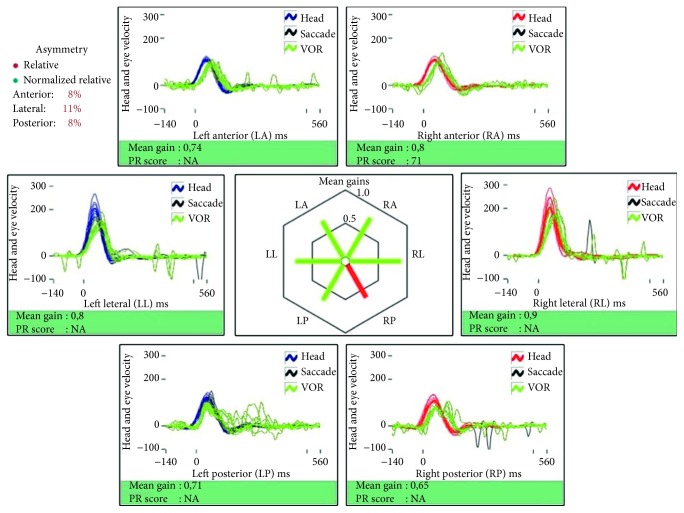
vIT 14 days after the beginning of the second episode shows complete recovery of gains.

In 2015, the patient suffered a new episode of acute vertigo. No infections had been reported during the weeks priorly. The clinical symptoms were similar, and he denied any hearing deterioration. The clinical examination was similar, and another vHIT showed a new decrease in superior and lateral semicircular canal gains (Figure 3) similar to that found in the first episode. Cervical and ocular vestibular evoked myogenic potentials (VEMPs) were performed with Fz vibration stimulation: cVEMPs showed a very low amplitude in both sides and oVEMPs were asymmetrical—the response below the left eye was significantly lower than the response below the right eye (interaural asymmetry ratio of 68%). Serum PCR for herpesviruses resulted negative, and there was no clinical evidence of other conditions which could be implicated. The patient was treated similarly. A new vHIT performed two weeks after the initiation of symptoms (Figure 4) showed a new recovery of the gains. At that time, neither spontaneous nor gaze-evoked or post-head-shaking nystagmus was objectified; MRI showed no gadolinium enhancement in the vestibule related to Meniere's disease.

The patient's background included prehypertension and left ventricular hypertrophy which did not change before or during both episodes of vertigo and were followed while he was admitted for medical treatment. He was also diagnosed with ocular sicca syndrome and vitiligo. He did not refer any previous episode or migraine manifestations. He was seen two years after the second episode and did not mention any vestibular symptom, and the clinical examination was normal.

## 3. Discussion

Vestibular neuritis is a frequent condition in otoneurological practice, but recurrence of this pathology is rare. This patient suffered a first episode of vestibular neuritis with quick and ad integrum recovery and a second ipsilateral event of similar characteristics from which also he recovered completely. No etiology could be demonstrated in either case.

In the scenario of a spontaneous acute vestibular syndrome, eye movement testing is highly accurate at disclosing a posterior fossa stroke, when applying the HINTS plus rule [13, 14]. The patient had a positive head impulse test for rightward head thrusts, and nystagmus was horizontal, direction fixed, and the slow phase directed toward the lowermost functioning side; in the alternating cover test, no ocular tilt was observed. There were no signs of hearing acuity reduction. In addition, his ability to stand and walk was tested, which has been proven to be an efficient ancillary clinical test to discriminate vestibular neuritis from stroke [15]. All these tests suggested right-side unilateral peripheral vestibulopathy. The patient was treated accordingly and evolved well; the MRI confirmed the absence of a significant vascular lesion and disclosed any other degenerative or tumoral etiology.

In both episodes, clinical signs and the findings in the vHIT provided sufficient data to localize the damage in the superior subdivision of the vestibular nerve. The characteristics of the bony canal which harbors the nerve in its intratemporal portion render it highly susceptible to entrapment and ischemia in case of swelling due to viral infection [16]. Absent response in the cVEMP is a frequent finding in elderly subjects in which a concomitant decline in high-frequency pure-tone average and amplitude of the cVEMP has been found [17]. In our patient, we can consider the existence of a precedent well-compensated bilateral saccular damage, as a high-intensity stimulation was used as recommended when (in elderly patients with chronic dizziness) absent cVEMP (acoustically evoked) is the only vestibular deficit found [18].

According to previous data, the patient was treated with steroids because of their anti-inflammatory properties and the major impact they have on early recovery, as opposed to their impact on long-term recovery of which no difference to natural course has been found [19–21]. Surprisingly, the patient developed a second episode that mimicked the first, not only with regard to clinical symptoms, but also in all measurements performed, raising the question of a viral hypothesis which is based on the previously mentioned anatomical characteristics of the superior vestibular nerve and also on recent “in situ hybridization” studies. They have shown that latent herpes simplex virus 1 infection in normal subjects is less frequent than first reported (18.4%) and that the neurons positive for HSV are located mainly in the superior vestibular nerve ganglion [22]. In our patient, there were no signs of herpes infection at the time of the vertigo episodes. Blood tests were negative, and he denied previous symptoms or signs related to herpes infection. This precluded prophylactic treatment with valacyclovir contrary to what has been recommended in a recurrent vestibular neuritis case that showed some similarities to ours [23].

It is remarkable that the recovery in the canal paresis occurred 12 days after the first episode. Recovery of canal function ranges from 50% to 70% after 10 years in some studies based on caloric testing [24, 25]. With the use of the vHIT, an interesting trend in recovery has been observed regarding etiology of the disease [23]. Other cases of recurrent vestibulopathy were ruled out, and the long-term evaluation (two years) revealed that the patient was doing well with no new vestibular symptoms.
